# Circulating RANKL is inversely related to RANKL mRNA levels in bone in osteoarthritic males

**DOI:** 10.1186/ar2348

**Published:** 2008-01-08

**Authors:** David Findlay, Mellick Chehade, Helen Tsangari, Susan Neale, Shelley Hay, Blair Hopwood, Susan Pannach, Peter O'Loughlin, Nicola Fazzalari

**Affiliations:** 1 Discipline of Orthopaedics and Trauma, University of Adelaide, North Terrace, Adelaide, 5000, Australia; 2 Hanson Institute, Frome Road, Adelaide, 5000, Australia; 3 Division of Tissue Pathology, Institute of Medical and Veterinary Science, Frome Road, Adelaide, 5000, Australia; 4 Division of Clinical Biochemistry, Institute of Medical and Veterinary Science, Frome Road, Adelaide, 5000, Australia; 5 Discipline of Pathology, University of Adelaide, North Terrace, Adelaide, 5000, Australia

## Abstract

**Introduction:**

The relationship of circulating levels of receptor activator of nuclear factor-κB ligand (RANKL) and osteoprotegerin (OPG) with the expression of these molecules in bone has not been established. The objective of this study was to measure, in humans, the serum levels of RANKL and OPG, and the corresponding levels in bone of mRNA encoding these proteins.

**Methods:**

Fasting blood samples were obtained on the day of surgery from patients presenting for hip replacement surgery for primary osteoarthritis (OA). Intraoperatively, samples of intertrochanteric trabecular bone were collected for analysis of OPG and RANKL mRNA, using real time RT-PCR. Samples were obtained from 40 patients (15 men with age range 50 to 79 years, and 25 women with age range 47 to 87 years). Serum total RANKL and free OPG levels were measured using ELISA.

**Results:**

Serum OPG levels increased over the age range of this cohort. In the men RANKL mRNA levels were positively related to age, whereas serum RANKL levels were negatively related to age. Again, in the men serum RANKL levels were inversely related (*r *= -0.70, *P *= 0.007) to RANKL mRNA levels. Also in the male group, RANKL mRNA levels were associated with a number of indices of bone structure (bone volume fraction relative to bone tissue volume, specific surface of bone relative to bone tissue volume, and trabecular thickness), bone remodelling (eroded surface and osteoid surface), and biochemical markers of bone turnover (serum alkaline phosphatase and osteocalcin, and urinary deoxypyridinoline).

**Conclusion:**

This is the first report to show a relationship between serum RANKL and the expression of RANKL mRNA in bone.

## Introduction

Our understanding of the molecular biology of bone turnover has advanced considerably in recent years with the demonstration that the activated receptor activator of nuclear factor-κB ligand (RANKL)/RANKL receptor complex promotes osteoclast differentiation and activity [1]. Osteoprotegerin (OPG), a secreted member of the tumour necrosis factor (TNF) receptor superfamily, acts as a natural antagonist of RANKL [2]. The roles played by RANKL and OPG in bone have been confirmed in mouse models of under-expression and over-expression or of exogenous administration of these molecules. For example, deletion of the gene encoding RANKL gives rise to osteopetrosis and impaired tooth eruption caused by the absence of mature osteoclasts [3], whereas injection of soluble RANKL causes a rapid rise in serum calcium levels caused by enhanced generation of osteoclasts and activation of existing osteoclasts [4]. On the other hand, the antiresorptive action of OPG was discovered by virtue of the remarkable increase in bone density found in transgenic mice over-expressing OPG [5], and deletion of the gene encoding OPG causes severe osteoporosis in mice [6]. The relevance of RANKL expression in human bone was highlighted by our study [7], which showed that histomorphometric indices of bone remodelling, namely eroded surface/bone surface ratio (ES/BS) and osteoid surface/bone surface ratio (OS/BS), are strongly associated with expression of RANKL mRNA in normal human trabecular bone. These data suggest that RANKL mRNA levels in bone represent surrogate measures of RANKL protein levels and also provide direct evidence that RANKL is involved in human bone remodelling.

There is now abundant evidence that the ratio of RANKL to OPG locally in bone controls osteoclast formation and activity, although it is also clear that this can be modulated by the prevailing cytokine environment [8-10]. RANKL is expressed by osteoblasts and other cells of the mesenchymal lineage, including periosteal cells, chondrocytes and endothelial cells [11,12], and also by activated T cells [3,13]. A large number of factors have been identified that can modulate the expression of RANKL by osteoblastic cells, as was recently reviewed [14].

We [15] and others [16] have reported that RANKL-induced osteoclast formation may be dysregulated in several bone loss pathologies, such as periprosthetic osteolysis, rheumatoid arthritis and periodontal disease, in which cells other than osteoblasts may become the source of RANKL. In postmenopausal osteoporosis, the reduction in oestrogen levels may also remove an important control on RANKL action and decrease the synthesis of OPG [17].

RANKL and OPG circulate in blood and, since the development of sensitive assays to measure serum levels, serum RANKL and OPG measurements have been the subject of numerous studies seeking to relate these levels to various clinical conditions [14,18,19]. These studies have shown, for example, that serum OPG levels increase with age [20], pregnancy [21] and vascular disease [22], and decrease in multiple myeloma [23]. Less clear trends have been found with serum RANKL levels, but these are reported to increase in multiple myeloma and to predict survival in this disease [24]. Schett and coworkers [25] reported that serum RANKL levels provide an independent predictor of fragility fracture, such that individuals with low circulating RANKL levels exhibited the greatest risk for fracture. RANKL is expressed in three molecular forms: a trimeric transmembrane protein [4], as found on osteoblasts; a truncated ectodomain cleaved from the cell-bound form by enzymatic cleavage by sheddase(s), such as TNF-α convertase (TACE) and matrix metalloproteinase (MMP)-14 [26-28], to release a soluble form of the molecule similar to that produced by recombinant means [4]; and a primary secreted form, as produced by activated T cells [3]. The cellular source(s) and molecular species that contribute to circulating RANKL are currently unknown.

The aim of this study was to determine how the serum levels of OPG or RANKL relate to their corresponding levels in bone and to measures of bone turnover. To facilitate sampling of both bone specimens and blood, the study group chosen consisted of men and women undergoing surgery for total hip replacement, with the primary diagnosis being OA. Relative levels of OPG and RANKL in bone were determined, using as surrogates the corresponding levels of mRNA derived by real-time RT-PCR. Circulating levels of total RANKL and free OPG were determined using ELISA. Bone turnover was assessed in terms of histomorphometric parameters in bone contiguous with that used for the mRNA extraction, and by measuring biochemical markers of bone turnover. In men, but not in women, it was found that circulating total RANKL levels were inversely associated with bone levels of RANKL mRNA. Levels of RANKL mRNA in bone were also found to be related to bone structural parameters and bone turnover indices in the male group.

## Materials and methods

Samples were obtained from 40 patients (15 men aged 50 to 79 years, and 25 women aged 47 to 87 years) presenting for total hip replacement surgery for OA. The protocol required exclusion from the study of individuals with overt metabolic bone disease, including Paget's disease, metastatic bone disease and rheumatoid arthritis. Fasting serum was collected on the morning of surgery and used for assay for serum total RANKL, serum OPG, and the circulating bone markers alkaline phosphatase and osteocalcin. In addition, fasting urine was collected for measurement of urinary pyridinoline and deoxypyridinoline. During surgery, cancellous bone samples were collected, as described below.

Informed consent was obtained from all patients included in the study, with approval from the Royal Adelaide Hospital Research Ethics Committee (Protocol No. 030305, granted 14 March 2003). Consent for use of human material was obtained from each patient after a full explanation of the purpose and nature of the research and the procedures to be used.

### Serum RANKL and OPG assays

Serum total RANKL levels were determined in fasting sera, using a sandwich ELISA kit designed for the quantitative determination of total (free RANKL and RANKL complexed to OPG) soluble RANKL in serum (Immunodiagnostik, Bensheim, Germany). Because only a small fraction of circulating RANKL is unbound, measurement of total RANKL was considered to reflect better the tissue production of soluble RANKL. This assay has been described in detail by Hofbauer and colleagues [29], and those authors found a significant positive correlation between free serum RANKL and total serum RANKL. Serum OPG levels were determined using an ELISA that measures free OPG (Immunodiagnostik), as also described by Hofbauer and colleagues [29]. Both assays were used in accordance with the manufacturer's instructions.

### Human bone specimens

Proximal femur specimens were obtained at the time of total hip replacement surgery. Tube saw core biopsies (10 mm) were taken from the intertrochanteric (IT) region of the proximal femur of each OA patient. We previously showed [30] that although there are differences in bone remodelling at the IT site between osteoarthritic and nonosteoarthritic individuals, the differences are not as great as those between the subchondral bone and the IT site in osteoarthritic individuals. These samples were cut into two equal pieces, which were used for histomorphometry and for the extraction of RNA.

### Histomorphometry

The IT bone specimens were rinsed in fresh sterile phosphate-buffered saline and stored overnight in 70% ethanol, and further processed undecalcified through a graded series of ethanol concentrations over a period of 1 week; the samples were then placed in acetone overnight. The bone specimens were then infiltrated and embedded in methyl methacrylate. All bone blocks were trimmed and sectioned on a microtome (Leica SP 1600; Leica Microsystems Pty Ltd, North Ryde, NSW, Australia). Sections, 5 μm thick, were stained using the von Kossa silver method and counter-stained with haematoxylin and eosin to distinguish between mineralized bone, cellular components of the marrow and osteoid. Histomorphometry was performed using an ocular mounted 10 × 10 graticule at a magnification of 100×. Histological measurements yielded the following parameters: percentage bone volume fraction (BV/TV [%]), specific surface of bone (BS/BV [mm^2^/mm^3^]), trabecular number (number/mm), trabecular thickness (μm), trabecular separation (μm), percentage osteoid surface (OS/BS [%]) and percentage eroded surface (ES/BS [%]).

### RT-PCR

For RNA preparation, the trabecular bone samples were rinsed briefly in diethylpyrocarbonate-treated water (Sigma-Aldrich Pty Ltd, Castle Hill, NSW, Australia) and then separated into small fragments, using bone cutters. Total RNA was extracted using an existing RNA preparation protocol described previously [31]. Total RNA prepared using this method was of sufficient quality to be used directly for real-time RT-PCR. RNA concentration and purity (260/280 absorbance ratio) were determined by spectrophotometry. RNA integrity was confirmed by visualization on ethidium bromide stained 1% weight/volume agarose formaldehyde gels. First-strand cDNA synthesis was performed with 1 μg total RNA from each sample, using a first-strand cDNA synthesis kit with Superscript II (Invitrogen; Carlsbad, CA, USA) and 250 ng random hexamer primer (Geneworks, Adelaide, SA, Australia), in accordance with the manufacturer's instructions.

RANKL, OPG, and glyceraldehyde phosphate dehydrogenase (GAPDH) mRNA expression was analyzed by real-time PCR, using BioRad iQ SYBR Green Supermix (BioRad, Hercules, CA, USA) on a Rotor-Gene thermocycler (Corbett Research, Mortlake, NSW, Australia). The reactions were incubated at 94°C for 10 minutes for one cycle, and then 94°C (20 seconds), 60°C (RANKL and GAPDH) or 65°C (OPG) all for 20 seconds) and 72°C (30 seconds) for 40 cycles. This set of cycles was followed by an additional extension step at 72°C for 5 minutes. All PCRs were validated by the presence of a single peak in the melt curve analysis, and amplification of a single specific product was further confirmed by electrophoresis on a 2.5% weight/volume agarose gel. Primers were designed for each gene to span at least one intron to avoid contaminating amplification from genomic DNA. Primer sequences were as follows; GAPDH, forward: ACCCAGAAGACTGTGGATGG; GAPDH, reverse: CAGTGAGCTTCCCGTTCAG; OPG, forward: CTGTTTTCACAGAGGTCAATATCTT; OPG, reverse: GCTCACAAGAACAGACTTTCCAG; and RANKL, forward: CCAAGATCTCCAACATGACT; and RANKL, reverse: TACACCATTAGTTGAAGATACT. GenBank accession numbers are as follows: GAPDH, NM_002046; OPG, NM_002546; and RANKL, NM_003701.

PCR reactions were carried out in triplicate for each sample. Relative quantification of RANKL and OPG mRNA expression between samples was calculated using the comparative cycle threshold (C_T_) method (ΔC_T_; Anonymous, User Bulletin #2, ABI PRISM 7700 Sequence Detection System, 1997). Briefly, the formula X_N _= 2^-ΔC^_T _was used, where X_N _is the relative amount of target gene in question and ΔC_T _is the difference between the C_T _of the gene in question and the C_T _of the housekeeping gene, GAPDH, for a given sample.

### Statistical analysis

Regression analysis was used to examine the relationship between the histomorphometric variables and female and male age-related changes. Statistical analysis was performed using GraphPad Prism software (V4.00 for Windows; GraphPad Software, San Diego, CA, USA).

The critical value for significance was chosen as *P *< 0.05.

## Results

### Osteoprotegerin

**Figure 1:**
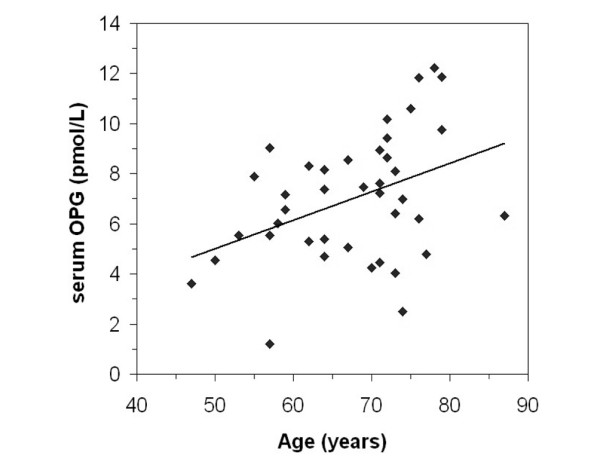
Serum OPG as a function of age in the pooled male and female groups. Fasting blood was taken at the time of operation for total hip replacement and serum osteoprotegerin (OPG) levels were determined in the men and women using ELISA and plotted as a function of age. Regression analysis indicated a positive correlation between serum OPG and age (*r *= 0.400, *P *= 0.01).

Mean serum free OPG levels were 7.4 pmol/l in both men and women (Table 1). A positive correlation was observed between fasting serum OPG levels and age, which was significant when data from men and women were pooled (*r *= 0.40, *P *= 0.01; Figure 1). An increase with age in a healthy adult population was previously reported [20]. In men, but not women, a significant association was found between bone OPG mRNA levels, measured using real-time RT-PCR, and serum OPG levels (*r *= 0.59, *P *= 0.028), although this was dependent on two extreme points. For neither men nor women was there a significant association between the OPG mRNA levels and RANKL mRNA levels, or between serum OPG levels and serum total RANKL levels. No significant relationships were observed between OPG mRNA, or serum OPG levels, and trabecular bone structural parameters, static indices of bone turnover, or circulating or urinary bone turnover markers.

**Table 1:** Structural parameters of trabecular bone, static indices of bone turnover and biochemical bone turnover measures

Parameter	Men	Women
BV/TV (%)	10.7 ± 4.0	9.8 ± 3.8
BS/BV (mm^2^/mm^3^)	22.1 ± 6.5	24.9 ± 8.5
Tb.N (number/mm)	1.1 ± 0.3	1.1 ± 0.3
Tb.Sp (μm)	900 ± 300	900 ± 300
Tb.Th (μm)	100 ± 30	100 ± 30
OS/BS (%)	5.6 ± 7.2	8.2 ± 10.5
ES/BS (%)	2.3 ± 1.9	2.0 ± 1.2
Serum total RANKL (pmol/l)	1,091 ± 781	1,688 ± 2471
Serum OPG (pmol/l)	7.4 ± 2.0	7.4 ± 2.8
Serum ALP (U/l)	92.9 ± 32.7	90.9 ± 19.7
Serum OCN (μg/l)	6.0 ± 3.7	5.8 ± 2.9
Urinary DPD (nmol/mmol creatinine)	18.3 ± 7.4	28.9 ± 7.5
Urinary PYR (nmol/mmol creatinine)	73.7 ± 27.7	102.7 ± 33.7

Results are expressed as mean ± standard deviation. ALP, alkaline phosphatase; BS/BV, specific surface of bone relative to bone tissue volume; BV/TV, bone volume fraction relative to bone tissue volume; DPD, deoxypyridinoline; ES/BS, eroded surface; OCN, osteocalcin; OPG, osteoprotegerin; OS/BS, osteoid surface; PYR, pyridinoline; RANKL, receptor activator of nuclear factor-κB ligand; Tb.N, trabecular number; Tb.Sp, trabecular separation; Tb.Th, trabecular thickness.

### Correlation between RANKL serum levels and RANKL mRNA expression in trabecular bone

**Figure 2:**
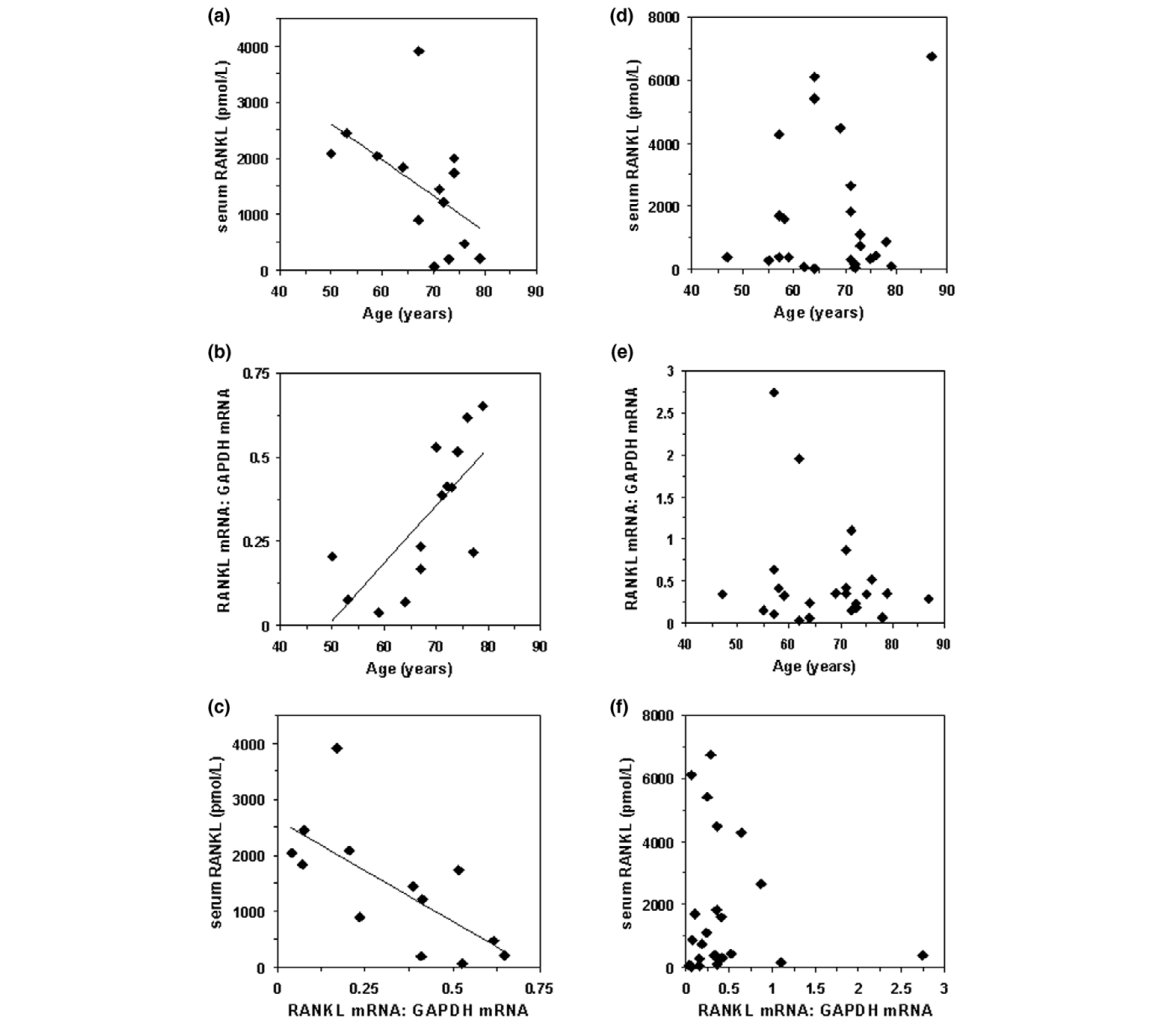
Serum RANKL and RANKL mRNA in cancellous bone from the proximal femur in men and women. **(a, d) **Fasting blood was taken at the time of operation for total hip replacement and serum total receptor activator of nuclear factor-κB ligand (RANKL) levels were determined, using ELISA, and plotted as a function of age. For the males (panel a), regression analysis indicated a negative correlation between these parameters (*r *= -0.52, *P *= 0.057; after removal of the outlier value: *r *= -0.67, *P *= 0.012). **(b, e) **Cancellous bone from the proximal femur was obtained at the time of operation for total hip replacement and extracted for RNA. RANKL mRNA levels, normalized against glyceraldehyde phosphate dehydrogenase (GAPDH) mRNA levels, were determined using real-time RT-PCR and are plotted as a function of age. For the men (panel b), regression analysis indicated a positive correlation between these parameters (*r *= 0.73, *P *= 0.003). **(c, f) **Serum total RANKL levels plotted against normalized RANKL mRNA levels. For the males (panel c), regression analysis indicated a negative correlation between these parameters (*r *= -0.70, *P *= 0.007). For the females (panels d, e and f), no correlations were found between any of these parameters.

Serum total soluble RANKL levels were determined for fasting sera, using a sandwich ELISA kit designed for quantitative determination of total (free RANKL and RANKL complexed to OPG) soluble RANKL in serum. The rationale for measuring total RANKL, rather than free RANKL, was that only a small fraction of circulating RANKL is unbound, and total RANKL was therefore considered to reflect better the tissue production of soluble RANKL. This assay was described in detail by Hofbauer and coworkers [29]. The mean fasting serum total RANKL levels were 1,091 pmol/l in the male group and 1,688 pmol/l in the women, with wide variance. These levels are approximately 1,000-fold higher than free serum RANKL, because most RANKL is complexed with OPG in serum [29]. Total serum RANKL levels were found to be negatively related to age in the men (*r *= -0.52, *P *= 0.057; without outlier: *r *= -0.67, *P *= 0.012; Figure 2a). RANKL mRNA levels, measured using real-time RT-PCR in RNA samples extracted from bone of the proximal femur, were found to be positively related to age in the male group (*r *= 0.73, *P *= 0.003; Figure 2b). When serum RANKL levels were plotted with bone RANKL mRNA levels, a significant negative correlation was identified (*r *= -0.70, *P *= 0.007; Figure 2c). No such relationships were found in analyses of the corresponding data for women; neither serum RANKL nor bone RANKL mRNA levels were found to be significantly associated with age (Figure 2d,e), and the two parameters were not significantly related to each other (Figure 2f).

### Correlations between serum RANKL, RANKL mRNA and bone structural and turnover parameters

The structural parameters of trabecular bone and the static indices of bone turnover, determined using histomorphometric analysis, and biochemical measures of bone turnover were similar in the male and female groups (Table 1). Consistent with these data, we previously reported no difference in the parameters BV/TV, BS/BV, trabecular separation, trabecular thickness and OS/BS in bone from the same intertrochanteric site from men and women older than 50 years [32]. In the previous study ES/BS was significantly lower in the women, which we did not observe in the cohort described here. The group included in our previous study were not known to have suffered from any disease affecting the skeleton.

We investigated relationships of bone RANKL mRNA levels and serum RANKL levels with trabecular bone structural parameters, static indices of bone turnover and biochemical markers of bone turnover. Table 2 shows the *r *values for these relationships in men, which indicate that the RANKL mRNA levels, or in some cases the ratio of RANKL mRNA/OPG mRNA, associated significantly with the other parameters. Because RANKL mRNA levels in bone appear to predict the corresponding levels of RANKL protein [7], the relationships that were observed are consistent with the known pro-resorptive role for RANKL in bone. Thus, RANKL mRNA levels were inversely related to the amount of trabecular bone, as indicated by BV/TV, and with the thickness of the trabeculae. On the other hand, RANKL mRNA was positively associated with BS/BV – a parameter that reflects a less plate-like trabecular structure, consistent with the effect of resorption in trabecular bone to create a more complex bone surface.

**Table 2:** Associations between bone RANKL mRNA levels and serum RANKL levels and other listed parameters

Parameter	Males		Females	
	
	Versus RANKL mRNA (versus RANKL/OPG mRNA; *r *[P value])	Versus serum RANKL (r [*P *value])	Versus RANKL mRNA (*r*)	Versus serum RANKL (*r*)
BV/TV	-0.53 (0.052)	0.10 (0.745)	-0.04	-0.20
BS/BV	0.57 (0.034)	-0.30 (0.296)	-0.12	-0.03
Tb.Th	-0.67 (0.011)	0.34 (0.230)	0.09	-0.05
ES/BS	(0.705 [0.003])	-0.35 (0.216)	0.33	-0.07
OS/BS	(0.80 [0.0003])	-0.02 (0.935)	0.28	0.03
ALP	(0.64 [0.011])	-0.49 (0.075)	0.07	0.08
OCN	0.71 (0.006)	-0.31 (0.283)	-0.02	-0.25
DPD	(0.85 [<0.0001])	-0.08 (0.785)	0.23	-0.25

Shown are correlations between receptor activator of nuclear factor-κB ligand (RANKL) mRNA levels (or RANKL mRNA/osteoprotegerin [OPG] mRNA) in bone and serum RANKL levels, and various structural parameters, static indices of bone turnover and biochemical bone turnover markers. ALP, alkaline phosphatase; BS/BV, specific surface of bone relative to bone tissue volume; BV/TV, bone volume fraction relative to bone tissue volume; DPD, deoxypyridinoline; ES/BS, eroded surface; OCN, osteocalcin; OS/BS, osteoid surface; Tb.Th, trabecular thickness.

In terms of the static indices of bone turnover, there were significant positive relationships between the RANKL/OPG mRNA ratio and ES/BS and OS/BS, which is consistent with our previous findings [7]. The finding of relationships between RANKL (or RANKL/OPG) and these parameters is consistent with the resorptive role of RANKL in bone and with the coupling between bone resorption and formation. The circulating bone turnover markers alkaline phosphatase, osteocalcin and urinary deoxypyridinoline were positively associated with RANKL mRNA and/or the RANKL/OPG mRNA ratio, which is consistent with these markers in turn relating to the extent of bone turnover. The relationships we observed were despite the fact that the mRNA analyzed using PCR was derived from a small discrete site at the proximal femur, whereas the circulating and urinary markers represented systemic bone turnover. Table 2 also shows that the relationships between serum RANKL and the histomorphometric and biochemical parameters were weaker than those found for RANKL mRNA levels. Importantly, however, the direction of the relationship between serum RANKL levels and the other parameters was in each case the inverse of that for RANKL mRNA. This remarkable finding is consistent with, and supports the validity of, the negative relationship found between serum RANKL and RANKL mRNA.

In contrast to the many strong relationships identified between bone mRNA levels and parameters of bone structure and turnover observed in the male cohort, the corresponding female data exhibited no significant relationships (Table 2). This was consistent with the lack of relationship found between serum RANKL and RANKL mRNA in the female cohort.

## Discussion

Many reports have described circulating levels of RANKL and OPG in health and disease [14], but the physiological significance of these parameters has not been established. In the present report we provide evidence linking circulating levels of these molecules to their levels in bone and to bone morphology. Interestingly, the relationships held only for a male cohort and not for an age-matched group of females.

Fasting serum OPG levels were found to correlate with age in this study if data from both the men and women were used. Significant correlations have been described for serum OPG with age in both men and women [33], although this is most easily observed in an extended age range from young adult to elderly. In another study of older men (from age 40 years), simple correlation analysis failed to show this relationship [34], although multiple regression analysis identified age as an independent predictor of serum OPG level. In the present study there was a significant link between bone OPG mRNA levels and serum OPG levels. This may indicate that bone is a major contributor to serum OPG or that OPG is similarly regulated at the mRNA level in other OPG producing tissues.

To our knowledge, this is the first report to show a relationship between serum RANKL and the expression of RANKL mRNA in the bone microenvironment. In males, the total levels of serum RANKL were negatively associated with age, and RANKL mRNA levels in bone were positively associated with age. Thus, total serum RANKL associated negatively with RANKL mRNA levels in bone. Providing strong support for this potentially important finding were the relationships that emerged between RANKL mRNA and measures of bone structure and turnover, parameters that are obtained by means very different from mRNA analysis. Indeed, in the men, RANKL mRNA levels and the ratio of RANKL/OPG mRNA were found to be associated with bone structure and turnover indices as well as with circulating and urinary bone turnover markers. The corresponding relationships with serum RANKL levels did not themselves reach statistical significance, but in each case the relationship with serum RANKL level was the inverse of the RANKL mRNA relationship. This is consistent with and supports the inverse relationship found between serum RANKL and RANKL mRNA levels in bone.

Several questions are raised by this study. The first is why were the relationships found between RANKL mRNA and serum RANKL levels and the other parameters in men not observed in women? This difference is not yet understood, although it is possible that the factors that drive bone turnover in this group of women aged 50 to 80 years are different from those in men of the same age range, because the female group included both early and late postmenopausal women. In addition, we have obtained other evidence that is consistent with molecular and biochemical differences between men and women with OA. In a gene microarray-based study, performed using similar bone samples to those analyzed in the present study, we identified clear sex-based differences in expression of a number of genes, including those encoding Wnt-5B and MMP-25, between cohorts of men and women with OA [35]. It is potentially important that the individuals investigated in the study had end-stage OA, because it has been reported that bone turnover is elevated in early OA [36]. Also, many of the individuals had at least one co-morbidity, such as hypertension or cardiovascular disease, and increased serum OPG has been reported to be associated with coronary artery disease [37]. However, the effects of OA and ageing were factors pertaining to both sexes. It would be ideal, although clearly impractical, to study a younger, premenopausal group of women in order to eliminate these confounding factors.

The second question raised by the data is what is the mechanism that gives rise to the inverse relationship between RANKL mRNA and serum RANKL observed in the men? Because we measured total serum RANKL, this cannot be accounted for by differential complexing of serum RANKL with OPG or other serum proteins. A possible explanation involves shedding of cell surface RANKL to release soluble RANKL into serum, and that this shedding activity is somehow decreased with increasing expression of RANKL mRNA. It has been shown that a number of TNF superfamily proteins can be released from the plasma membrane, a process termed 'ectodomain shedding' [38,39]. TNF-α is cleaved at the cell surface by the metalloprotease-disintegrin TACE [38], and RANKL can be cleaved by one or more sheddase activities, initially reported to be TACE or a related protease [26]. We previously reported that treatment of primary human osteoblasts with zoledronic acid increased their expression of TACE, with concurrent reduction in the cell surface expression of RANKL [40]. The TACE inhibitor TAPI-2 partially restored cell surface RANKL expression, suggesting that TACE and possibly additional metalloproteinases may be involved in the cleavage of transmembrane RANKL in human osteoblasts.

Recent evidence suggests that MMP-14 (membrane type 1 MMP) may be an important RANKL sheddase in the mouse, perhaps regulated by OPG [41]. MMP-14 and ADAM-10 (a disintegrin and metalloprotease-10) have been shown to have strong RANKL shedding activity in that species, and suppression of MMP-14 in primary mouse osteoblasts increased cell membrane-bound RANKL and increased the ability of these cells to promote osteoclastogenesis in co-culture with macrophages [28]. Interestingly, marked reduction in release of soluble RANKL by osteoblasts deficient in MMP-14 was observed, and serum level of RANKL in MMP-14 deficient mice was reported to be undetectable. In human osteoblasts, parathyroid hormone (PTH) treatment concurrently increased the expression of RANKL mRNA and decreased MMP-14 production [42]. It was proposed that the decreased MMP-14 expression by PTH may lead to RANKL-induced activation of osteoclasts by increasing the local concentration of cell surface RANKL. This study is potentially relevant to our observations because it has been well documented that serum PTH levels increase with advancing age (reviewed by Portale and coworkers [43]). Although we did not measure serum PTH levels in the present study, the effect of age on RANKL levels that we observed, at least in men, might be accounted for by increased PTH levels with age. Further studies are required to resolve these mechanistic issues. We found that MMP-14 mRNA is abundantly expressed in human bone, but the molecule clearly plays several roles in the skeleton [44].

With respect to our observation of an inverse relationship between serum RANKL and bone RANKL mRNA, it is interesting that a study conducted in postmenopausal women with fragility fracture [25] showed that the women in the highest tertile for serum RANKL had the lowest risk for fracture, whereas those in the lowest tertile had the highest risk for fracture. Although that study has been criticized on a number of grounds, including small numbers of fractures [45], the relationship between serum RANKL and fracture risk might warrant closer examination in the light of our findings. Significantly, we previously reported increased expression of RANKL mRNA in trabecular bone from individuals with osteoporotic fracture of the proximal femur [46].

## Conclusion

The present study provides evidence for relationships between serum RANKL and RANKL expressed in bone and between bone RANKL mRNA levels and bone turnover processes. These relationships were only identified in a male cohort, and further work will be required to determine why this might be different in women. The findings suggest that serum RANKL may be useful as a bone turnover marker, initially in men, and prompts further investigation of mechanisms. For example, there may be a role of RANKL sheddases in regulating RANKL activity in human bone, perhaps providing another level of regulation to OPG.

## Abbreviations

BS/BV = specific surface of bone relative to bone tissue volume; BV/TV = bone volume fraction relative to bone tissue volume; C_T _= cycle threshold; ELISA = enzyme-linked immunosorbent assay; ES/BS = eroded surface/bone surface ratio; GAPDH = glyceraldehyde phosphate dehydrogenase; MMP = matrix metalloproteinase; OA = osteoarthritis; OPG = osteoprotegerin; OS/BS = osteoid surface/bone surface ratio; PTH = parathyroid hormone; RANKL = receptor activator of nuclear factor-κB ligand; RT-PCR = reverse transcription polymerase chain reaction; TACE = tumour necrosis factor-α convertase; Tb.N = trabecular number; TNF = tumour necrosis factor.

## Competing interests

The authors declare that they have no competing interests.

## Authors' contributions

DF contributed to the design of the project, raised funding for the work, directed the project and was primarily responsible for writing the manuscript. MC contributed practically to the work in terms of acquisition of the samples at operation, raised funds for the work and provided intellectual input throughout, including critical revision of manuscript drafts. HT conducted a large amount of analysis of the results, including the histomorphometry and molecular analyses, and provided critical revision of manuscript drafts. SN and SP worked with the patients to obtain informed consent to take samples, organized the biochemical analysis, assisted with preparation of the figures for the manuscript and the references, and provided critical revision of manuscript drafts. SH, BH and PO'L also conducted biochemical and molecular analyses, assisted with data interpretation and provided critical revision of manuscript drafts. NF directed the histomorphometry and provided primary interpretation of the results, assisted in raising funds for the project and provided critical revision of manuscript drafts. All authors read and approved the final manuscript.
